# *Lactococcus garvieae*: An Uncommon Human Pathogen Causing Infective Endocarditis in a Valve-in-Valve Transcatheter Aortic Valve Replacement

**DOI:** 10.1155/2021/5569533

**Published:** 2021-07-11

**Authors:** Diego H. González-Bravo, Sergio Alegre-Boschetti, Richard Silva-Cantillo, Joshua Mercado-Maldonado, Reyshley Ramos-Márquez, Gabriel Torres-Rivera, Carlos Cortés, Josue Mercado-Crespo

**Affiliations:** ^1^Cardiovascular Division, Department of Medicine, VA Caribbean Healthcare System, San Juan, Puerto Rico; ^2^Internal Medicine Division, Department of Medicine, VA Caribbean Healthcare System, San Juan, Puerto Rico; ^3^Internal Medicine and Nephrology Division, Department of Medicine, VA Caribbean Healthcare System, San Juan, Puerto Rico

## Abstract

*Lactococcus garvieae* is a fish pathogen and an uncommon cause of human infections. There is a growing body of evidence showing its potential for causing endocarditis especially in those with prior valve surgery. In this case report, we present what we believe is the first case of endocarditis by *L. garvieae* affecting a valve-in-valve transcatheter aortic valve replacement that was successfully treated. Specific guidelines for the management of these patients are lacking. Our experience can contribute to the current knowledge regarding this life-threatening infection as well as to the future care of these patients. We aim to emphasize that despite not being recognized as a typical endocarditis microorganism by the Duke Criteria, the possibility of endocarditis needs to be highly entertained in patients with *L. garvieae* bacteremia, especially when prosthetic valves are present. Consequently, clinicians should pursue further this diagnosis with transesophageal echocardiogram and/or alternative imaging modalities (e.g., PET-CT scan and MRI) regardless of an initial negative transthoracic echocardiogram. Reaching a diagnosis of *L. garvieae* endocarditis led us to the decision of prolonging the antibiotic course for 6 weeks with successful results. Ultimately, surgery was not required owing to the absence of prosthetic aortic valve dysfunction and paravalvular extension of the infection.

## 1. Introduction

**Figure 1:**
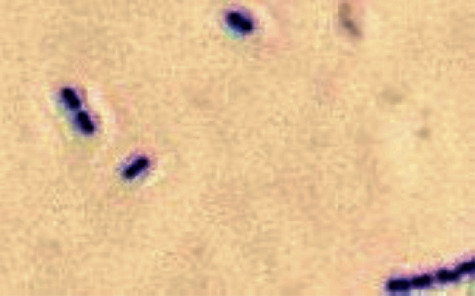
Microscopy. Gram-stain smear (1000× magnification) showing gram-positive (violet) cocci in chains consistent with *Lactococcus garvieae.*

*Lactococcus garvieae* is a gram-positive coccus (Figure 1) and catalase-negative bacterium known for producing fish infection outbreaks in warm waters (rainbow trout) [1–3]. Its zoonotic potential was unknown until recently when multiple case reports have shown the spectrum of pathogenic capabilities of this bacterium, capable of causing urinary tract infections, liver abscess, and most importantly infective endocarditis (IE) [3–6].

Human consumption of raw seafood seems to be the main contamination source; nonetheless, infection from unpasteurized dairy products has also been described [2–4]. Other risk factors for infection are an immunosuppressed state, prior heart valve surgery, and the presence of gastrointestinal diseases that compromise the mucosal lining integrity (polyps, diverticulosis, peptic ulcers, and gastroesophageal reflux disease [GERD]) [2, 4].

We present a case that we believe is the first instance of *L. garvieae* endocarditis in a valve-in-valve transcatheter aortic valve replacement (TAVR). We aim to raise awareness about the potential for endocarditis of this blooming human pathogen as we discuss its diagnosis and our treatment experience.

## 2. Case Presentation

### 2.1. History of Present Illness

A 78-year-old man who presented with a one-day history of fever, chills, and altered mental status (AMS) preceded by 7 days of malaise and anorexia. At the emergency room (ER), he was found normotensive (107/67 mmHg), afebrile (37.1°C), not hypoxic (98%), and without tachypnea (19 rpm), yet tachycardia (131 bpm) was present. He was disoriented and lethargic but showed no focal neurological deficit nor signs of meningeal irritation. His heart rhythm was irregular, and a systolic ejection murmur (II/VI) was present at the right upper sternal border. Jugular venous distension was not evident, and his extremities were warm and without edema. His abdomen was nontender. Lungs were clear to auscultation, and no skin stigmata for endocarditis was appreciated (splinter hemorrhages, Osler, and Janeway lesions). Recent surgeries, new medications, travels, and toxic habits were all denied.

### 2.2. Past Medical History

His medical history was remarkable for essential hypertension, dyslipidemia, GERD, atrial fibrillation (afib) on chronic anticoagulation (apixaban 5 mg twice per day), and a bioprosthetic surgical aortic valve replacement (SAVR) in 2008 followed by valve-in-valve TAVR (2017) two years prior to presentation. Dietary habits included regular consumption of raw salmon.

### 2.3. Investigations

**Figure 2:**
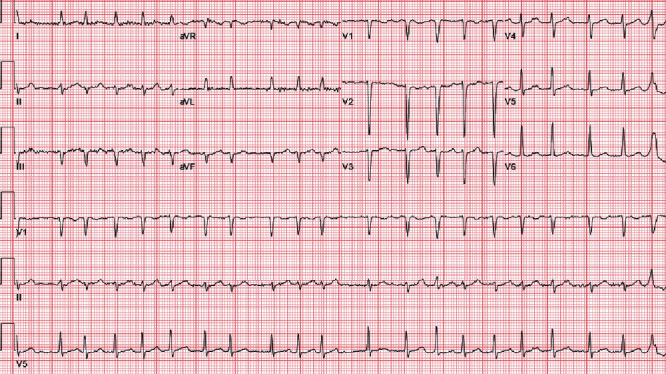
Electrocardiogram. Initial electrocardiogram revealing atrial fibrillation with fast ventricular response (126 bpm). There were no new ST-segment changes suggestive of acute ischemia.

**Figure 3:**
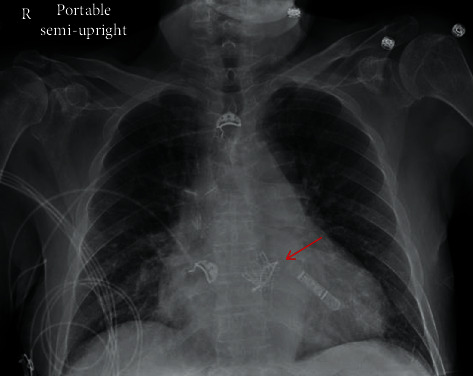
Chest X-ray (CXR): there are mildly increased interstitial markings (chronic in character), cardiomegaly, and no evidence of consolidation or pleural effusion. Loop monitor can be visualized in the CXR at the left lower chest wall as well as a valve-in-valve TAVR (red arrow).

Initial electrocardiogram (Figure 2) showed afib with a fast ventricular response (FVR), without chest pain or new ST-segment changes compelling for an acute coronary syndrome. Chest X-ray (Figure 3) and head CT scan were negative for acute pathologies. However, mild leukocytosis (11.3 × 10^3^/*μ*L) with neutrophilia, elevated ESR (72 mm/1Hr), CRP (214.7 mg/L), and procalcitonin levels (0.20 ng/mL) were present, all suggestive of an underlying bacterial infection. His toxicology, urinalysis, and HIV tests were unremarkable.

### 2.4. Differential Diagnosis and Management

His encephalopathy in the context of afib was worrisome for stroke; however, no focal neurological deficits were found, plus his head CT scan was negative, and he had been compliant with anticoagulation therapy. His laboratories and tachycardia suggested an underlying bacterial infection of unclear foci. At first, meningoencephalitis was contemplated although meningeal irritation signs were missing. A lumbar puncture was attempted but ultimately deferred as the patient became aggressive and uncooperative with the procedure. As per the sepsis protocol, the patient was pan-cultured, resuscitated with intravenous (IV) fluids, and started on broad-spectrum IV antibiotics (vancomycin and cefepime). Acyclovir was added given the possibility of herpetic meningoencephalitis.

**Figure 4:**
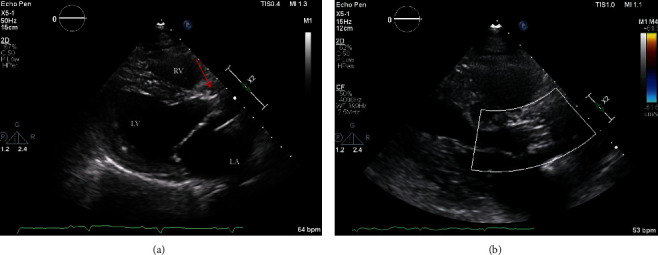
Transthoracic echocardiogram parasternal long-axis view. (a) Valve-in-valve TAVR (red arrow) with some artifacts causing suboptimal resolution and visualization of the bioprosthesis, yet no overt large vegetations were detected. No pericardial effusion present and systolic function was preserved (55%). (b) Diastolic frame showing no evidence of significant regurgitation by color Doppler. RV: right ventricle; LV: left ventricle; LA: left atrium.

**Figure 5:**
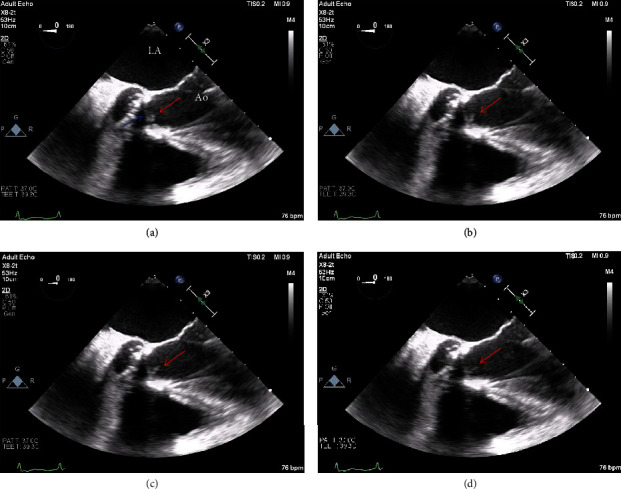
Transesophageal echocardiogram (TEE) midesophageal views (Video 1). Various frames of TEE images showing an elongated and oscillating echogenic structure (red arrow) attached to the right coronary cusp leaflet of the valve-in-valve TAVR (blue arrow) consistent with an infective endocarditis vegetation (measuring 0.7 cm). No significant insufficiency or paravalvular extension of infection was detected. No evidence in favor of valvular thrombosis. The other native valves (especially the mitral valve) had no vegetations. LA: left atrium; Ao: ascending aorta.

Subsequently, blood cultures (4/4) grew *Lactococcus garvieae* (Figure 1). Transthoracic echocardiogram (TTE) revealed no prosthetic aortic valve dysfunction nor large vegetations (Figure 4). Nonetheless, a transesophageal echocardiogram (TEE) showed a pedunculated/oscillating echo-dense structure measuring 0.7 cm in its largest dimension attached to the right coronary cusp of the TAVR consistent with vegetation (Figure 5, Video 1). There were no cusp perforations, significant valvular insufficiencies, paravalvular abscess or leaks, nor evidence of valve thrombosis (normal valve hemodynamics by Doppler, plus valve was bioprosthetic and not mechanical, and patient was compliant with chronic anticoagulation).

## 3. Discussion

Prosthetic valve endocarditis (PVE) accounts for only 10-30% of all cases of infective endocarditis (IE) with an incidence of 1-6%, affecting the TAVR and SAVR population to a similar extent [7, 8]. It is believed to be the worst presentation of IE, a life-threatening infection of the lining of the heart (endocardium) that can lead to systemic embolization, valve destruction, heart failure, and death [9]. Most cases of PVE are caused by the typical endocarditis microorganisms: *Staphylococcus aureus* or coagulase-negative staphylococci. Nevertheless, in our patient, it was surprisingly caused instead by *Lactococcus garvieae*, a fish pathogen. Our patient's GERD and regular consumption of raw salmon were risk factors for infection.

*Lactococcus garvieae* is becoming a blooming human pathogen. Its potential for causing endocarditis has been unraveling for the last few years [5, 6]. There are less than 30 cases reported in the available literature, this one being the ninth case involving an aortic valve replacement (AVR), the sixth case reported in the USA, the first one documented in the island of Puerto Rico, and the first one affecting a valve-in-valve TAVR [6]. Multiple cases (20% = 5/25) have also been described in the Asian continent [6]. The incidence and prevalence remain unknown, plus it is believed to be underdiagnosed since misclassification of this pathogen as other gram-positive cocci (enterococci and streptococci) has been commonly described [5]. In our case, mass spectroscopy (MALDI-TOF MS) provided the identity of this unusual pathogen; however, 16 rRNA PCR has also been used successfully [1, 5].

Malek et al. evaluated 25 cases of *L. garvieae* IE and showed that infection affected more commonly older men (median age = 68 years) and most frequently presented as subacute fever with chills (median = 14 days) [5]. A history of valve replacement (8/25 aortic, 5/25 mitral) was identified in 52% of these patients, for which 54% (7/13) of them had evidence of vegetations associated to their prosthetic valves. The native mitral valve (64%) was the most infected valve, even in cases where an AVR was present. Moreover, *L. garvieae* IE seems to have profound implications in terms of morbidity and mortality. The fatality rate was 16%, and half of the patients had complications such as valve dehiscence/rupture, septic emboli, heart failure, stroke, shock, or renal failure. Furthermore, 48% of cases required surgery.

An appropriate diagnosis of IE is done so by using the Duke Criteria (DC) that proposes a definite diagnosis in those with bacteremia and imaging evidence of valvular involvement (vegetations and paravalvular abscess) [9]. Initially, our patient had an unremarkable TTE and unfulfilled DC for definitive endocarditis (only 3 minor criteria were present: atypical microorganism, fever and predisposition/AVR). We were initially hesitant to perform a TEE as this was not a typical endocarditis microorganism (staphylococci and yeast) for which the probability of endocarditis is known to be high. Nevertheless, discovering the growing evidence of IE surrounding this pathogen pushed us to perform the TEE, which provided the major criteria that was missing in order to establish a definitive diagnosis of IE (1 major + 3 minor). Consequently, we want to emphasize that since the risk of endocarditis in these patients seems to be high, it is reasonable to pursue an in-depth evaluation with TEE or alternative imaging modalities (e.g., MRI and PET-CT) before ruling out such diagnosis after an initial negative TTE [9].

Arriving at this diagnosis changed drastically the management of our patient. First, by extending his antibiotic course to 6 weeks instead of providing him with only 2 weeks, as indicated for uncomplicated bacteremia, and second, it prompted cardiothoracic surgery evaluation.

There is no standardized treatment of *L. garvieae* endocarditis due to lack of randomized control trials (RCT), but it has been treated successfully with beta-lactams (ampicillin 2 g q4hrs or ceftriaxone 2 g q12-24 hrs), or vancomycin (30 mg/kg/day divided in 2-3 doses), with or without aminoglycosides (gentamycin 3 mg/kg/day) for 6 weeks [5, 6, 9]. Whether surgery is always required for the treatment of these patients remains elusive.

In general, indications for PVE surgical intervention follow the same principles as for native valve endocarditis [7]. Guidelines recommend a heart team-based approach to this decision. Patients who benefit the most from surgery are the ones with highly virulent pathogens (staphylococci or fungi), those with prosthetic valve dysfunction (severe regurgitation, paravalvular abscess/fistula, heart block, dehiscence, and perforation) causing heart failure, persistent bacteremia (after 5-7 days of antibiotics), recurrent embolic events, or vegetations larger than 1 cm [7, 9]. Our patient had none of these complications and since he responded positively to antibiotic therapy with rapid clearance of bacteremia plus subsequent TTE remain essentially unremarkable, the decision was taken in conjunction with a cardiothoracic surgeon to continue conservative management.

## 4. Follow-Up

**Figure 6:**
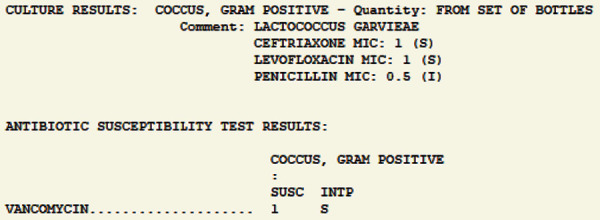
*Lactococcus garvieae* antimicrobial sensitivities.

Pathogen susceptibility (Figure 6) allowed for de-escalation of antibiotics to ceftriaxone (2 g q12 hrs) achieving sterile blood (hospitalization day #2) and resolution of tachycardia and AMS. He was discharged and completed 6 weeks of IV ceftriaxone at a skilled nursing facility without complications.

## 5. Conclusions

The possibility of endocarditis needs to be highly entertained when confronting patients with *L. garvieae* bacteremia, especially in the presence of prosthetic heart valves. Therefore, despite an initial negative TTE, the clinician should seek an in-depth evaluation with TEE and/or alternative imaging modalities (e.g., PET-CT, MRI) in view that these patients seem to have a high risk for endocarditis. A missed diagnosis could be fatal since about half of these patients can develop complications and require open-heart surgery. However, we presented a case of *L. garvieae* endocarditis, which we believe is the first one affecting a valve-in-valve TAVR that was successfully treated conservatively and did not develop complications that required surgery possibly due to early identification.

## Data Availability

The data and test results used in this case report are all included in this article.

## Abbreviations

Afib: Atrial fibrillation
AMS: Altered mental status
AVR: Aortic valve replacement
FVR: Fast ventricular response
GERD: Gastroesophageal reflux disease
IE: Infective endocarditis
MALDI-TOF MS: Matrix-assisted laser desorption/ionization time-of-flight mass spectrometry
MRI: Magnetic resonance imaging
PET-CT: Positron emission tomography-computed tomography
PVE: Prosthetic valve endocarditis
TAVR: Transcatheter aortic valve replacement
TEE: Transesophageal echocardiogram
TTE: Transthoracic echocardiogram.

## Supplementary Materials

Supplementary Materials Video 1: Transesophageal echocardiogram (TEE). Midesophageal and short-axis views of the valve-in-valve TAVR showing an elongated and oscillating echogenic structure measuring 0.7 cm attached to the right coronary cusp leaflet consistent with an infective endocarditis vegetation. https://drive.google.com/file/d/19ot-M103npHVx4vp8QQJPKfDxp-_oURH/view?usp=sharing.

## References

[1] Navas M. E., Hall G., El Bejjani D. (2013). A case of endocarditis caused by Lactococcus garvieae and suggested methods for identification. *Journal of Clinical Microbiology*.

[2] Wang C. Y., Shie H. S., Chen S. C. (2007). Lactococcus garvieae infections in humans: possible association with aquaculture outbreaks. *International Journal of Clinical Practice*.

[3] Tariq E. F., Irshad Y., Khalil H. B., Khakwani A. S., Khan U. A. (2020). Urinary tract infection caused by the novel pathogen, Lactococcus garvieae: a case report. *Cureus*.

[4] Choksi T. T., Dadani F. (2017). Reviewing the emergence of Lactococcus garvieae: a case of catheter associated urinary tract infection caused by Lactococcus garvieae and Escherichia coli coinfection. *Case Reports in Infectious Diseases*.

[5] Malek A., De la Hoz A., Gomez-Villegas S. I., Nowbakht C., Arias C. A. (2019). Lactococcus garvieae, an unusual pathogen in infective endocarditis: case report and review of the literature. *BMC Infectious Diseases*.

[6] Rösch R. M., Buschmann K., Brendel L., Schwanz T., Vahl C. F. (2019). Lactococcus garvieae endocarditis in a prosthetic aortic valve: a case report and literature review. *Journal of Investigative Medicine High Impact Case Reports*.

[7] Habib G., Lancellotti P., Antunes M. J. (2015). 2015 ESC guidelines for the management of infective endocarditis: the task force for the management of infective endocarditis of the European Society of Cardiology (ESC). Endorsed by: European Association for Cardio-Thoracic Surgery (EACTS), the European Association of Nuclear Medicine (EANM). *European Heart Journal*.

[8] Butt J. H., Ihlemann N., de Backer O. (2019). Long-term risk of infective endocarditis after transcatheter aortic valve replacement. *Journal of the American College of Cardiology*.

[9] Baddour L. M., Wilson W. R., Bayer A. S. (2015). Infective endocarditis in adults: diagnosis, antimicrobial therapy, and management of complications: a scientific statement for healthcare professionals from the American Heart Association. *Circulation*.

